# Echinochrome A Protects against Ultraviolet B-induced Photoaging by Lowering Collagen Degradation and Inflammatory Cell Infiltration in Hairless Mice

**DOI:** 10.3390/md19100550

**Published:** 2021-09-28

**Authors:** Jung Eun Seol, Sang Woo Ahn, Bomin Seol, Hyeong Rok Yun, Nammi Park, Hyoung Kyu Kim, Elena A. Vasileva, Natalia P. Mishchenko, Sergey A. Fedoreyev, Valentin A. Stonik, Jin Han

**Affiliations:** 1 Department of Dermatology, Inje University Busan Paik Hospital, College of Medicine, Inje University, Busan 47392, Korea; derma09@hanmail.net (S.W.A.); illillil@naver.com (B.S.); 2 Department of Physiology, College of Medicine, Cardiovascular and Metabolic Disease Center, Smart Marine Therapeutic Center, Busan 47392, Korea; foryou018@naver.com (H.R.Y.); nammi780314@gmail.com (N.P.); estrus74@gmail.com (H.K.K.); 3 Department of Health Sciences and Technology, Graduate School, Inje University, Busan 47392, Korea; 4 G.B. Elyakov Pacific Institute of Bioorganic Chemistry, Far-Eastern Branch of the Russian Academy of Science, 690022 Vladivostok, Russia; vasilieva_el_an@mail.ru (E.A.V.); mischenkonp@mail.ru (N.P.M.); fedoreev-s@mail.ru (S.A.F.); stonik@piboc.dvo.ru (V.A.S.)

**Keywords:** echinochrome A, photoaging, hairless mice, dermal collagen degeneration, mast cell infiltration

## Abstract

Echinochrome A (Ech A, 7-ethyl-2,3,5,6,8-pentahydroxy-1,4-naphthoquinone) has been known to exhibit anti-oxidative and anti-inflammatory effects. However, no study has been carried out on the efficacy of Ech A against skin photoaging; this process is largely mediated by oxidative stress. Six-week-old male SKH-1 hairless mice (*n* = 36) were divided into five groups. Except for a group that were not treated (*n* = 4), all mice underwent ultraviolet-B (UVB) exposure for 8 weeks while applying phosphate-buffered saline or Ech A through intraperitoneal injection. UVB impaired skin barrier function, showing increased transepidermal water loss and decreased stratum corneum hydration. UVB induced dermal collagen degeneration and mast cell infiltration. Ech A injection was found to significantly lower transepidermal water loss while attenuating tissue inflammatory changes and collagen degeneration compared to the control. Furthermore, Ech A was found to decrease the relative expression of matrix metalloproteinase, tryptase, and chymase. Taken together, these results suggest that Ech A protects against UVB-induced photoaging in both functional and histologic aspects, causing a lowering of collagen degradation and inflammatory cell infiltration.

## 1. Introduction

**Figure 1:**
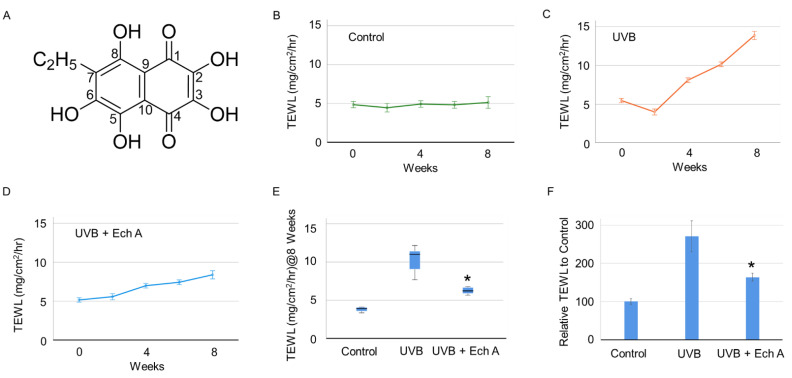
Changes in skin physiological function over time in the dorsal skin of UV-irradiated mice and age-matched non-treated mice. (**A**) The chemical structure of Ech A; (**B**–**F**) Histogram showing the transition of TEWL among the control (**B**), UVB (**C**), and UVB + Ech A (**D**) groups over 2 months of the experiment. Ech A injection induced a significantly low level of TEWL compared to the UVB group (**E**–**F**). *, *p* < 0.05 vs. UVB.

Echinochrome A (Ech A) is a quinoid pigment (Figure 1A) in the polyhydroxynaphthaquinone family that is extracted from sea urchin shells or spines [1]. The moiety of Ech A is known to have anti-oxidative and anti-inflammatory effects [2]. In Russia, Echi A has been used as an ingredient in medication called Histochrome^®^, showing protective effects against oxidative stress in cardiovascular or ocular degenerative disorders [3,4,5]. In other studies, Ech A has been shown to reduce the level of mitochondrial reactive oxygen species (ROS) in cardiomyocytes [6], exert anti-ulcerogenic effects in a gastric ulcer model [7], attenuate oxidative stress and pro-inflammatory cytokine secretion in an acute uveitis model [8], and correct imbalances in the intestinal immune system in an inflammatory bowel disease model [9]. Furthermore, several clinical trials have supported the efficacy of Ech A in various diseases such as ophthalmic, cardiovascular, cerebrovascular, inflammatory, metabolic, and malignant diseases [10]. However, so far there has been no study of the relationship between the anti-oxidative effect of Ech A and skin aging. 

Skin aging is caused by a combination of intrinsic factors, such as genetic factors and hormonal changes, and extrinsic factors, including ultraviolet (UV) radiation, cigarette smoking, and mechanical stress [11]. Along with chronological aging, solar UV irradiation has been noted as a crucial component of skin aging [12]. Skin exposed to UV produces a large amount of ROS, which react with deoxyribonucleic acid (DNA), proteins, and fatty acids, resulting in oxidative damage. Oxidative stress initiates a signaling pathway cascade, which leads to the induction of activator protein 1 (AP-1) and the down-regulation of transforming growth factor beta (TGF-β), followed by the expression of metalloproteinase (MMP) and the inhibition of collagen synthesis [11,13,14]. The accumulation of metabolic disruption is manifested as morphological features of photoaged skin, such as deep wrinkles, sagging, laxity, and uneven pigmentation [12,15].

Many studies have been conducted on natural extracts in order to investigate the candidates for anti-aging products based on the anti-oxidative properties of chemicals including flavonoids, polyphenols, carotenoids, and vitamins C and E [13,14,16,17]. Plant extracts such as soy bean [18], sea buckthorn [19], turmeric, green tea, grape, soybean, milk thistle, pomegranate [20], Equisetum arvens [21], Gastrodia elata [22], Nymphaea tetragona [23], Euterpe oleracea, Matricaria chamomilla, and Camellia sinensi [24] have been widely studied. Although topical and oral applications of these plant extracts have been researched, there have been few studies dealing with skin aging and the anti-oxidative effects of marine bioactive compounds [25,26,27,28,29].

In this study, we investigated the in vivo therapeutic potential of Ech A against UVB-induced photoaging in a murine model. The effects of Ech A in terms of the aspects of gross visual skin condition, skin barrier function, and histologic analysis were evaluated.

## 2. Results

### 2.1. Effects of Ech A on the Skin Physiological Function

The values of transepidermal water loss (TEWL) and stratum corneum hydration (SCH) were measured every two weeks in the dorsal skin of mice. There was no significant difference in TEWL and SCH in week 2 between any of the groups. However, a significant elevation of TEWL and a decrease in SCH were observed in UVB-irradiated mice from week 4 (Figure 1B–D). The UVB irradiation only group (UVB) showed a more rapid elevation of TEWL compared to the UVB irradiation + Ech A-treated group (UVB + Ech A) until the end of the experiment (*p* < 0.05) (Figure 1C,D). At the end of the experiment at week 8, TEWL was significantly higher in the UVB group compared to the UVB + Ech A group (Figure 1E,F). 

**Figure 2:**
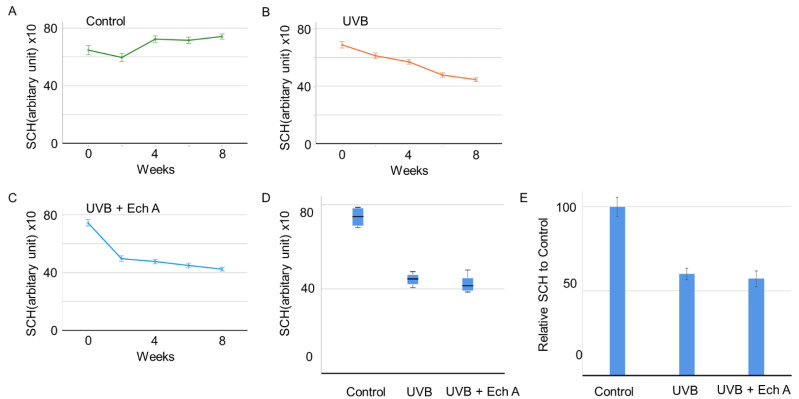
Changes in skin physiological function over time in the dorsal skin of UV-irradiated mice and age-matched non-treated mice. (**A**–**E**) Histogram showing the transition of SCH among the control (**A**), UVB (**B**), and UVB + Ech A (**C**) groups over 2 months of the experiment. There was no significant protective effect of Ech A on SCH compared to the UVB group (**D**,**E**).

Similarly, from week 4, a significant decrease in SCH was observed in UVB-irradiated mice (Figure 2A–C). However, there was no significant difference between the UVB and UVB + Ech A groups in terms of the SCH value at the end of the experiment (Figure 2D).

### 2.2. Effects of Ech A on the Macroscopic Appearance of the Mouse Skin

**Figure 3:**
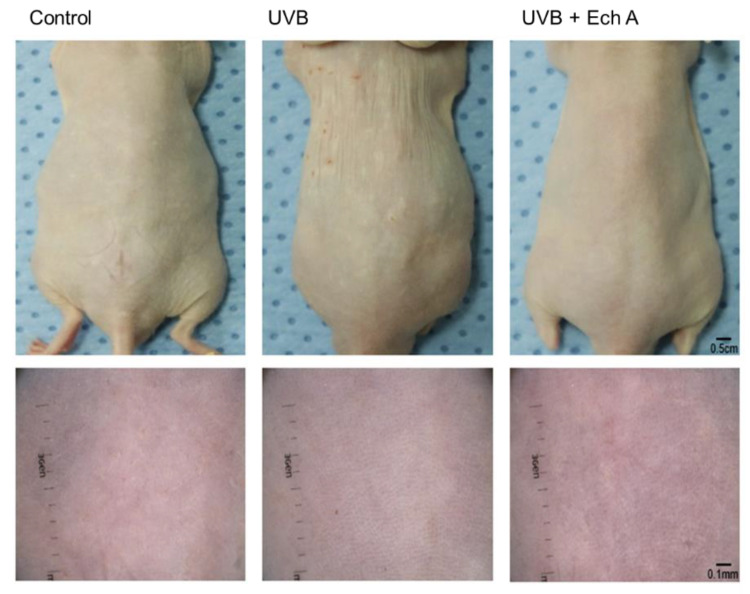
Macroscopic observation in the dorsal skin of UV-irradiated mice and age-matched non-treated mice. UV irradiation induced cutaneous changes, including superficial wrinkles, irregular pigmentation, and telangiectasia. There was no significant macroscopic difference between the UVB and UVB + Ech A groups.

On the day after the completion of the experiment, mice were observed and photographed using a camera and dermoscopy. Upon gross inspection, it could be seen that the dorsal skin of the UV-irradiated groups featured more fine superficial wrinkles, hypopigmented macules, or yellowish discoloration compared to the control group. Similar features were observed under dermoscopic inspection, showing more pronounced crossing wrinkles, irregular pigmentation with hypopigmentation and yellowish dots, and fine telangiectasia. However, there was no significant difference in gross cutaneous findings between the UVB and UVB + Ech A groups (Figure 3).

### 2.3. Effects of Ech A on the Histopathologic Features of the Mouse Skin

**Figure 4:**
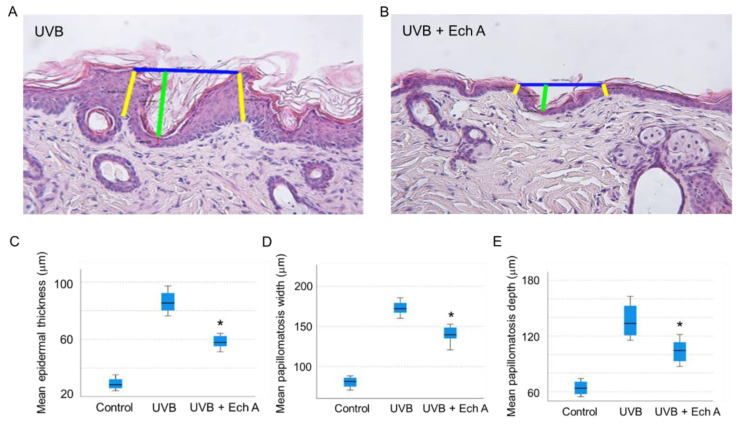
Histometry and statistical analysis of epidermal thickness and papillomatosis width and depth in the dorsal skin of mice. (**A**,**B**) The histometry of the site is as mentioned in the description of the method. The epidermal thickness (yellow line) and papillomatosis width (blue line) and depth (green line) of a mouse were calculated as mean values from five different sites on the H&E slides of each mouse (H&E, ×200). (**C**–**E**) The mean epidermal thickness and papillomatosis width and depth were all significantly higher in the UVB group than in the control group. There was significant decrease in the mean epidermal thickness and papillomatosis width and depth in the UVB + Ech A group compared to the UVB group. * *p* < 0.05 vs. UVB.

Upon histopathologic examination of the dorsal skin tissue, it could be seen that the UVB group exhibited prominent changes in both the epidermis and dermis. In the epidermis, the UVB group showed more severe hyperkeratosis, acanthosis, spongiosis, and papillomatosis than the UVB + Ech A group (Figure 4A,B).

In particular, the mean epidermal thickness (Figure 4C), papillomatosis width (Figure 4D) and papillomatosis depth (Figure 4E) were significantly increased in the UVB group compared to the UVB + Ech A group, measured as the mean value of five randomly selected areas of each H&E tissue slide.

**Figure 5:**
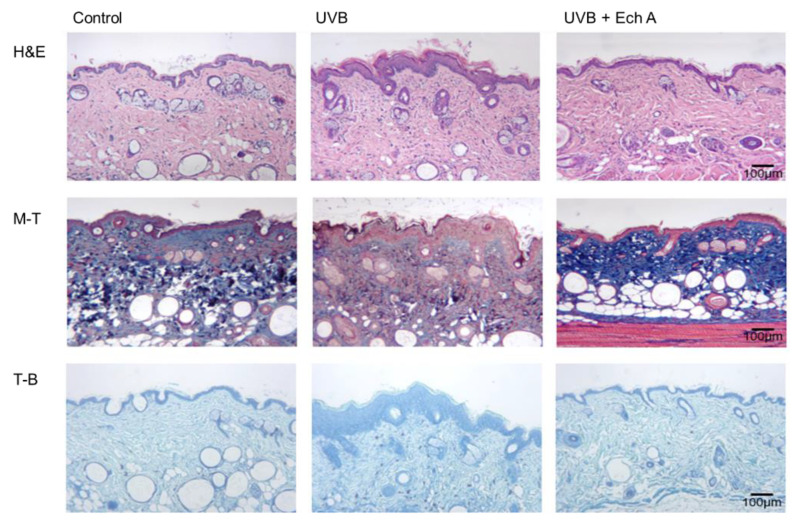
Histological observation of the dorsal skin of UV-irradiated mice and age-matched non-treated mice. Significant epidermal changes, such as hyperkeratosis, acanthosis, spongiosis, and papillomatosis, were observed. Dermal changes were accompanied by perivascular and interstitial inflammatory cell infiltration. Epidermal and dermal changes were more evident in the control than in the group treated with Ech A. In the Masson’s trichome (M-T, ×100) staining, a significant decrease in dermal collagen fibers was observed in the UVB compared to the UVB + Ech A group. Furthermore, toluidine blue (T-B, ×100) staining showed more dense mast cell infiltration in the UVB group than the UVB + Ech A-treated group.

In the dermis, the UV irradiation induced perivascular, interstitial inflammatory cell infiltration mostly composed of lymphocytes. Upon inspection of H&E slides, the UVB group showed significantly denser inflammatory cell infiltration and epidermotropism compared to the UVB + Ech A group, featured by lymphocyte exocytosis, spongiosis, and subsequent proliferation of the epidermis. Furthermore, the distribution and amount of dermal collagen fiber were diffuse and dense in the UVB + Ech A group, whereas the UVB group showed a marked decrease in collagen fiber. Toluidine blue stain showed more dense infiltration of mast cells in the UVB group than the UVB + Ech A group (Figure 5). 

**Figure 6:**
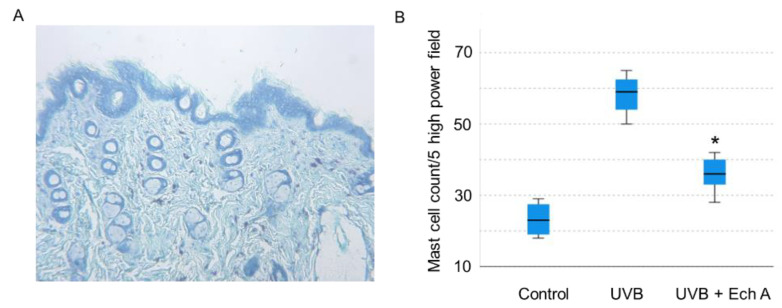
Histologic appearance of dorsal skin (**A**, toluidine blue, ×100) and statistical analysis of mast cell count (**B**). UVB irradiation induced a significant increase in the mast cell count, while the UVB group showed more dense mast cell infiltration compared to the UVB + Ech A group. *, *p* < 0.05 vs. UVB.

The number of mast cells was counted in sum of 5 high power field in each toluidine-blue stained slide (Figure 6A). Mean mast cell count was significantly higher in the UVB group than in the UVB + Ech A-treated group (Figure 6B). 

### 2.4. Effects of Ech A on the Immunohistochemical Expression Levels of Collagen 1,3, MMP 1,2,9 and Beta-Galactosidase of the Mouse Skin

**Figure 7:**
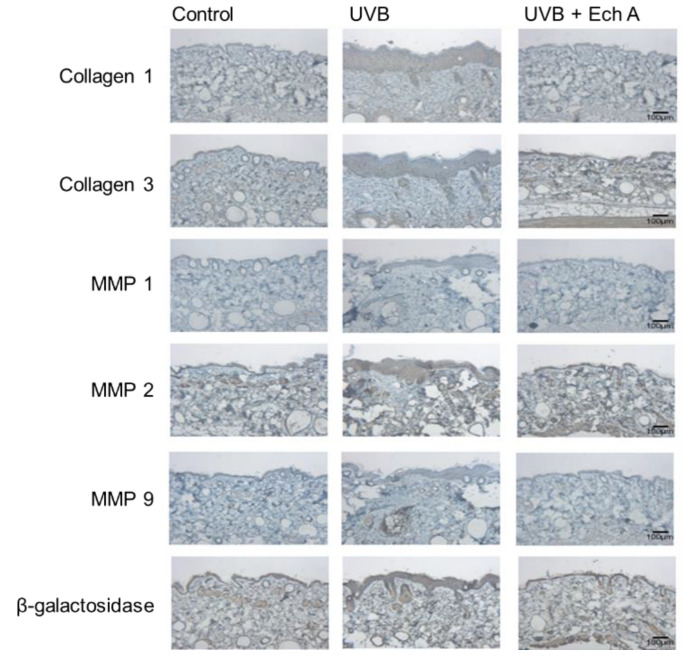
Immunohistochemical staining of biopsy specimen of the dorsal skin of mice. Biopsy specimens were stained with anti-collagen 1,3 antibodies, anti-MMP 1, 2, 9 antibodies, and beta-galactosidase antibody. Higher amounts of collagen 1 and 3 were observed in the UVB + Ech A group than in the UVB group, while higher amounts of MMP 1, 2, and 9 were observed in the UVB than in the UVB + Ech A group. Beta-galactosidase stained all groups similarly (×100 magnification).

Immunohistochemical staining was conducted on the dorsal skin of mice with six antibodies, including anti-collagen 1,3 antibody, anti-MMP 1,2,9 antibodies, and beta-galactosidase antibody. The staining results for each molecule showed different distribution among the three groups. Higher amounts of collagen 1 and 3 were observed in the UVB + Ech A group than in the UVB group, whereas MMP 1, 2, and 9 were more densely stained in the UVB group compared to the UVB + Ech A group. However, there was no significant difference in beta-galactosidase staining among all the groups (Figure 7).

### 2.5. Effects of Ech A on the Expression of Matrix Metalloproteinase, Tryptase, and Chymase of the Mouse Skin

**Figure 8:**
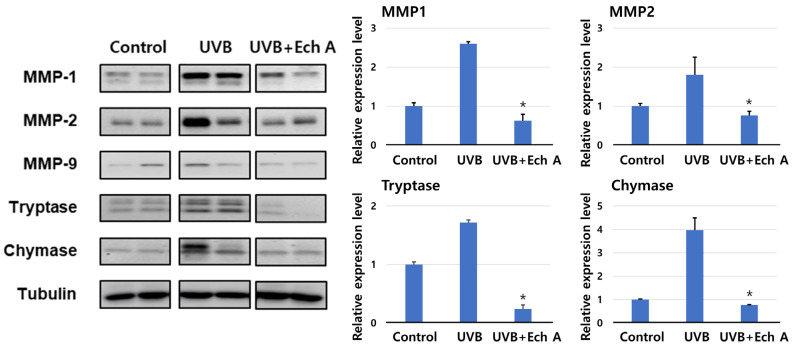
Western blotting of biopsy specimen of the dorsal skin of mice. Western blotting showed increased expression of MMP 1, 2, tryptase, and chymase in the UVB group compared to the control and the UVB + Ech A groups. * *p* < 0.05 vs. UVB.

The relative expressions of MMP 1, 2, tryptase, and chymase were significantly increased in the UVB group compared to the control and the UVB + Ech A groups (Figure 8). However, there was no significant difference in expression between the control and the UVB + Ech A groups (Figure 8). Although the expression of MMP 9 was slightly higher in the UVB group, it was too trivial to compare the expression among all groups.

## 3. Discussion

Skin undergoes two distinct types of aging. Intrinsic skin aging is caused by inevitable physiologic changes, including a weakened intrinsic antioxidant defense system, proteasome activity, and telomerase function [11]. It is characterized by dry and pale skin with fine wrinkles and increased laxity. In contrast, extrinsic skin aging, also called photoaging, includes the preventable structural and functional changes that are mostly induced by unprotected ultraviolet exposure [12]. Typical features of photoaging are deep wrinkles, laxity, coarseness, increased fragility, and multiple telangiectasia. Both types of aging share a common pathophysiology of oxidative damage, which triggers downstream events in the proinflammatory pathway, including changes in nuclear factor κB, interleukin-1 and 6, vascular endothelial growth factor, and tumor necrosis factor-α. This is followed by the deterioration of intracellular proteins, the induction of matrix-degrading metalloproteins, and DNA damage, including the disruption of the normal loop structure at the end of telomeres [11,12,13,14,15].

In the process of photoaging, UVB is the primary source of direct DNA damage and inflammation. UVB (280 to 320 nm) can penetrate the epidermis and upper portion of the dermis, while UVA (320 to 400 nm) can deeply penetrate the skin down to the lower dermis. In addition to the common mechanism of aging, UV damage contributes to further collagen degradation and the production of elastotic material in the skin [30,31]. Photoaging is also associated with mitochondrial DNA mutation and decreased metabolic function through the generation of reactive oxygen species [30,31]. Furthermore, UV irradiation also induces basement membrane disruption and accumulation of matrix metalloproteinases and urinary plasminogen activator in the skin [32]. The fragmentation of collagen due to degradation is crucial step in the clinical appearance of wrinkle formation [33].

Numerous studies dealing with protection against photoaging have been carried out. The anti-inflammatory and anti-oxidative properties of various molecules, including flavonoids, polyphenols, carotenoids, and vitamins C and E, and natural extracts, have been investigated with regard to their ability to prevent or stimulate collagen degeneration or synthesis [13,14,18,19,20,21,22,23,24]. However, no previous study exploring the therapeutic potential of marine bioactive material in skin photoaging has been carried out.

Ech A, which is a natural quinoid pigment in the polyhydroxynaphthaquinone family that is extracted from sea urchin shells or spines, has been studied regarding its anti-oxidative abilities through the moiety of metal chelator [2]. In previous studies, Ech A was found to activate the transcription of genes responsible for mitochondrial biogenesis in vitro and modulate mitochondrial respiration in the cardiomyoblast H9c2 cell line and isolated rat cardiomyocytes [6]. Furthermore, Ech A has shown anti-inflammatory properties in studies of gastric ulcer, acute uveitis, and inflammatory bowel disease models [6,7,8,9]. Based on its anti-oxidative and anti-inflammatory properties and efficacy in mitochondrial functional improvement, Ech A has been speculated to have therapeutic potential against photoaging, considering its common pathophysiology characterized by oxidative stress-induced proinflammatory cytokine secretion and collagen degradation. Therefore, this study investigated the effect of Ech A on UV-induced skin photoaging in structural and functional aspects using a hairless mouse model.

As the effect of Ech A on the skin has not been studied so far, little data are available regarding the extent of the systemic absorption of Ech A through oral intake. Consequently, methods of intraperitoneal injection were adopted in this study and further research is needed to investigate the pharmacokinetics and pharmacodynamics of Ech A under variable routes of administration.

Transepidermal water loss (TEWL) and stratum corneum hydration (SCH) are widely adopted indexes used to assess skin barrier function [34,35]. In this experiment, UVB exposure was found to significantly increase TEWL and decrease SCH in both the UVB and the UVB + Ech A groups. This result indicates the clinical effect of UVB exposure on the deterioration of general skin barrier function. In particular, the extent of TEWL elevation was attenuated by Ech A treatment, implying the protective effect of Ech A on skin barrier function. Although SCH was not found to be significantly different between the UVB and the UVB + Ech A groups, it is speculated that the experimental period was too short to fully display the long-term effect of improved skin barrier function on moisture retention. A more prolonged period of study is necessary in order to evaluate the precise correlation between skin barrier function and stratum corneum hydration state in hairless mice. Similarly, an extended period of observation is necessary in order to detect changes in macroscopic appearance caused by any histologic improvement.

The histopathologic effect of UV irradiation has been well studied and the hairless mouse is known to express various epidermal and dermal changes similar to that of human skin [34]. Continuous UV irradiation can induce epidermal and dermal changes in hairless mouse skin, including hyperkeratosis, acanthosis, dermal perivascular inflammatory cell infiltration, the excessive deposition of the abnormal elastin complex, and (most importantly) the impairment of collagen fibers [34,36,37]. Collagens 1 and 3 are crucial components of tissue tensile strength, being abundantly distributed in the dermis and polymerized to extended fibrils [38]. Therefore, it is widely accepted that dermal collagen density and distribution are responsible for the wrinkle formation in photoaged skin [33,39,40].

In this study, UVB irradiation caused significant histopathologic changes similar to those seen in previous studies, showing marked hyperkeratosis, proliferation of the Malpighi layer, spongiosis, papillomatosis, perivascular and interstitial inflammatory cell infiltration, increased levels of mast cells, and decreased levels of collagen fibers. Ech A intraperitoneal injection attenuated all the histopathologic changes, showing less severe epidermal changes along with more compact collagen fiber distribution and a lower mast cell count. These protective effects of Ech A are speculated to be achieved by the anti-oxidative and anti-inflammatory mechanism through modulating mitochondrial metabolism, as in other studies involving cardiomyocytes, gastric mucosal cells, or eye corneal cells [6,7,8,9].

Matrix metalloproteinases (MMPs) are zinc-binding proteolytic enzymes that mediate extracellular matrix degradation and tissue remodeling [41]. Various types of MMPs including MMP 1, 2, 3, and 9 share a common molecular structure and degrade specific ECM substrates [42]. Based on this property, MMPs are widely used as useful markers for severe photoaging. In particular, MMP2 and 9 are the main gelatinases increased by UV irradiation in a hairless mouse model, degrading the dermal collagen and basement membrane [43,44]. In addition, MMP 1 acts as a crucial proteinase in human skin photoaging and has also been suggested to be a molecule regulating interleukin-1α and β induced by UV irradiation in a mouse model [45]. Ech A was found to reduce the amount of MMPs in both immunohistochemistry and Western blotting, indicating that the clinical efficacy of Ech A originates from the reduced collagen degradation and dermal inflammatory cell recruitment, including mast cells, rather than from stimulating collagen synthesis.

Beta galactosidase is another indicator of photoaging [46]; however, no significant change was found after the application of Ech A. It is possible that Ech A may not target the beta galactosidase-associated pathway, or that the expression of beta galactosidase could be low in hairless mice. The use of a longer schedule of experiments might induce significant differences in beta galactosidase.

## 4. Materials and Methods

### 4.1. Preparation of Ech A

Histochrome^®^ containing 1% Ech A (PN002363/02) was obtained from the Pacific Institute of Bioorganic Chemistry, Far East Branch of the Russian Academy of Sciences. The composition of Histichrome is 1% of Ech A in sodium carbonate and sodium chloride 0.9% isotonic solution and the concentration is 37.5 mM. It was kept in refrigerator at 2 °C.

### 4.2. Care and Use of Animals

This study was conducted with the approval of scientific and ethical standards given by Inje University Institutional Animal Care and Use Committee (IU-IACUC; approval number 2020-002). The animals used in the experiment were six-week-old SKH-1 hairless mice purchased from Orient Bio Laboratory Animals (Dae jeon, Korea). All mice were raised in 12 h light/dark cycles at a temperature of 22 ± 2 ℃ and 45 ± 5% humidity and given free access to a standard irradiated chow diet and water.

### 4.3. Experimental Design

All mice (n = 20) were assigned to one of three groups: a not-treated group (not treated; control, n = 4) and two UV radiation groups. The UV radiation groups were subdivided into a phosphate-buffered saline (PBS) intraperitoneal injection group (UVB, n = 8) and an Ech A intraperitoneal injection group (UVB + Ech A, n = 8).

### 4.4. UV Irradiation

A UV irradiation device was manufactured by NSC, Inc. (Hwasung, Korea) in the shape of a rectangular box that was 60 cm wide, 40 cm long, and 40 cm high. The device was equipped with a TL20W/01RS lamp (Phillips™, Eindhoven, Netherlands) that emitted UVB with a narrow peak wavelength of 311 nm. The distance between the UV lamp and the mouse was maintained at 30 cm, and the amount of UVB emitted was measured by an ILT-1700 Research Radiometer (International Light Technology™, Boston, MA, USA). The eight-week schedule of irradiation included irradiation every two days, with the mice resting for two days after every third radiation given each week. The UVB was given at the amount of 1 minimal erythema dose (1 MED) during the first week, gradually rising to 2 MED, 3 MED, and 4 MED during the next 3 weeks, and then reaching a plateau at 4 MED until the end of the 8th week.

### 4.5. Setting 1 MED

MED refers to the minimum UV irradiation amount that can cause erythema on the skin. MED is generally used as a practical indicator for determining the degree of effects of UV irradiation. In order to set the level of 1 MED, a cloth with a hole 1 cm in diameter was used to cover the dorsal skin of the mouse, and six different areas were irradiated with 60, 80, 100, 120, 140, and 160 mJ. The amount of UVB at the height of mouse was calculated as about 1 mJ per second, so setting MED took about 11 min of irradiation. After 24 h, it was observed whether erythema had occurred in any of the six points.

### 4.6. Intraperitoneal Injection

Immediately after UVB irradiation, the UVB and UVB + Ech A groups were injected with the reagents in the intraperitoneal space. UVB group was administrated with PBS 200 μL. Ech A dose in UVB + Ech A group was 0.1 mg/kg of Ech A mixed with PBS 200 μL.

### 4.7. Clinical Observation of skin Condition

In order to check the change in the dorsal skin conditions, photographs were taken with a camera and dermoscope (DermLite™ DLCAM, Dermlite, San Juan Capistrano, CA, USA) before every first irradiation day of each week. Mice were photographed under anesthesia, which was administered through inhaling isoflurane briefly.

### 4.8. Measurement of Transepidermal Water Loss, Stratum Corneum Hydration

The day after the completion of every 2 week schedule of UV irradiation, the transepidermal water loss (TEWL) and stratum corneum hydration (SCH) were measured by a tewameter and corneometer using a Cutometer™ MPA 580 (Courage and Khazaka Electronics™, Cologne, Germany). A measuring probe was placed on the middle portion of the dorsal skin of the mice, which were anesthetized by inhaling a low concentration of isoflurane. Values of TEWL were read at the plateau of the estimation graph, while SCH values were taken as the mean value of three measurements.

### 4.9. Histological Analysis

Two days after the last UV irradiation, all mice were sacrificed using CO_2_ gas to collect the dorsal skin tissue samples. Collected samples were cut into four pieces and one of them was fixed with 4% paraformaldehyde solution at room temperature for 24 h, then transported in 50% ethyl alcohol solution. After another 24 h, the samples were embedded in paraffin wax and sectioned into 4 µm slices. The skin sections were stained with hematoxylin and eosin (H&E), Masson’s trichrome, and toluidine blue. Immunohistochemistry staining was conducted with anti-collagen I antibody, anti-MMP9 antibody, and anti-beta galactosidase antibody purchased from Abcam^®^ (Cambridge, UK), as well as anti-collagen III polyclonal antibody, anti-MMP 1 polyclonal antibody, and anti-MMP2 monoclonal antibody purchased from Invitrogen™ (Carlsbad, CA, USA). Stained slides were observed and photographed by optical microscopy Olympus™ BH2 (Tokyo, Japan) and a Tucsen™ Digiretina 16 (Fuzhou, China). Epidermal thickness was measured in H&E-stained slides through the computer-assisted program Tucsen™ TCapture (Fuzhou, China). The mean epidermal thickness was calculated by measuring the distance between the dermoepidermal junction and the top of stratum granulosum at five different areas in each specimen at a magnification of ×200. In addition, papillomatosis width was measured as the horizontal distance between the shoulders of the papillomatosis, while depth was calculated as the shortest distance between the bottom point of the basal layer sulcus and the horizontal line between papillomatosis shoulders. In addition, the number of mast cells on a toluidine blue-stained slide was calculated as the mean value of 5 randomly chosen visual fields at ×100 magnification. 

### 4.10. Western Blot

Mouse dorsal skin tissues were lysed with radioimmunoprecipitation assay (RIPA) buffer (50 mM Tris-HCl, pH 7.5, 150 mM NaCl, 0.5% sodium deoxycholate, 1% Triton X-100, 2 mM EDTA, and 0.1% SDS) supplemented with protease and phosphatase inhibitors (200 mM PMSF). The protein concentration was analyzed using a Bio-Rad DC protein assay (Bio-Rad Laboratories, Inc., Hercules, CA, USA). The lysate was separated by 8~15% sodium dodecyl sulfate polyacrylamide gel electrophoresis (SDS-PAGE) and transferred onto a nitrocellulose membrane (GE Healthcare, Chicago, IL, USA) for 100 min at 100 V at 4 °C. Membranes were blocked with 5% skim milk in TBST (20 mmol/L Tris-HCl, pH 7.5, 50 mmol/L NaCl, and 0.1% Tween 20). After blocking, the membranes were incubated for overnight with primary antibodies diluted to 1:1000 in 3% bovine serum albumin (BSA) in TBST. After washing 3 times for 15 min, the membranes were incubated for 1 h with horseradish peroxidase-labeled secondary antibody diluted to 1:5000 in 3% BSA. The protein bands were visualized using an enhanced chemiluminescence kit (Santa Cruz Biotechnology, Dallas, TX, United States), then observed with an AI 600 Imager (GE Healthcare, Chicago, IL, USA).

### 4.11. Statistical Analysis

Using the SPSS program (version 10.0; SPSS Inc., Chicago, IL, USA), continuous variables were presented as averages with standard errors. Correlation analysis, *t*-test, and repeated measures ANOVA methods were used to determine the association between each factor, and regression or logistic regression were used. A *p*-value of <0.05 was considered statistically significant.

## 5. Conclusions

In this study, Ech A showed a protective effect against UVB-induced photoaging in both functional and structural aspects. It is estimated to be induced by the anti-oxidative and anti-inflammatory properties of Ech A, affecting the mitochondrial metabolism. Inhibitory effects on mast cells and MMP might contribute to histologic differences, such as attenuated epidermal thickening, lower levels of inflammatory cell infiltration, and preserved collagen fiber, followed by the ultimate improvement of the skin barrier function. 

To the best of our knowledge, this is the first study dealing with the effect of Ech A on skin photoaging in a murine model. It is possible that there are other contributory signal pathways or cytokines such as transcription factor activation protein 1 or nuclear factor kappa B. To compensate for the limitations of this study, it is necessary to determine the precise molecular mechanism or signal cascade pathways on variable target cells through further research.

## Data Availability

This article does not data and the data availability policy is not applicable to the article.
